# Human β-defensin 3 has immunosuppressive activity *in vitro* and *in vivo*

**DOI:** 10.1002/eji.200940041

**Published:** 2010-01-26

**Authors:** Fiona Semple, Sheila Webb, Hsin-Ni Li, Hetal B Patel, Mauro Perretti, Ian J Jackson, Mohini Gray, Donald J Davidson, Julia R Dorin

**Affiliations:** 1 MRC Human Genetics Unit, Institute of Genetics and Molecular MedicineEdinburgh EH4 2XU, Scotland, UK; 2 Centre for Inflammation Research, QMRI University of EdinburghUK; 3 William Harvey Research Institute, Barts and The London School of Medicine, Queen Mary University of LondonUK

**Keywords:** Anti-inflammatory, cAMP, Defensin, TNF-α

## Abstract

β-defensins are antimicrobial peptides with an essential role in the innate immune response. In addition β-defensins can also chemoattract cells involved in adaptive immunity. Until now, based on evidence from dendritic cell stimulation, human β defensin-3 (hBD3) was considered pro-inflammatory. We present evidence here that hBD3 lacks pro-inflammatory activity in human and mouse primary Mφ. In addition, in the presence of LPS, hBD3 and the murine orthologue Defb14 (but not hBD2), effectively inhibit TNF-α and IL-6 accumulation implying an anti-inflammatory function. hBD3 also inhibits CD40/IFN-γ stimulation of Mφ and *in vivo*, hBD3 significantly reduces the LPS-induced TNF-α level in serum. Recent work has revealed that hBD3 binds melanocortin receptors but we provide evidence that these are not involved in hBD3 immunomodulatory activity. This implies a dual role for hBD3 in antimicrobial activity and resolution of inflammation.

## Introduction

β-defensins are broad spectrum, cationic, antimicrobial peptides. They are expressed predominantly at mucosal surfaces and believed to be important components of innate immunity although their precise *in vivo* role has not been clarified 1. Human β-defensins are a multigene family and the main cluster on chromosome 8p23 has been shown to be copy number variable 2. Increased copy number in humans is associated with psoriasis and decreased copy number with Crohn's disease, suggesting involvement in these autoimmune diseases 3, 4. Human β defensin-3 (hBD3) is one of the most cationic of the β-defensins with broad spectrum, salt insensitive, antimicrobial activity 5. It is highly expressed in psoriatic skin and the reproductive tract 6, 7. Defensins have been considered pro-inflammatory as their expression increases in response to TLR ligands, TNF-α, IL-1β, IFN-γ and PMA, and following infection or injury 5, 8, 9. In addition, they have been shown to chemoattract CD4 T cells and immature dendritic cells through CCR6, suggesting that they link innate and adaptive immunity 10. hBD3 and 4 also chemoattract monocytes and Mφ 8, 11, and hBD3 has been shown to activate monocytes and myeloid dendritic cells through TLR-1/2 by inducing expression of co-stimulatory molecules and NF-κB 12. Recently, human α-defensins present in neutrophil granules have been shown to display anti-inflammatory properties 13.

In this paper we show that hBD3 does not induce TNF-α or IL-6 in Mφ and in fact has potent anti-inflammatory effects on both human and mouse primary Mφ. The anti-inflammatory effect was also evident *in vivo* and in the THP-1 human monocytic cell line and RAW264.7 mouse Mφ cell line. hBD3 effectively inhibited the inflammatory effects of both LPS and CD40 ligand (CD40L). Recently it has been shown that hBD3 can interact with melanocortin receptors *in vitro* 14 and a dominant mutation in this gene in dogs and arctic wolves is causative for black coat colour 15. Despite melanocortin 1 receptor (MC1R) and melanocortin 3 receptor (MC3R) being expressed on Mφ and having known immunomodulatory activity, we show here that these receptors do not mediate the novel, potent anti-inflammatory effect displayed by hBD3.

## Results and discussion

### hBD3 is anti-inflammatory *in vitro*

**Figure 1:**
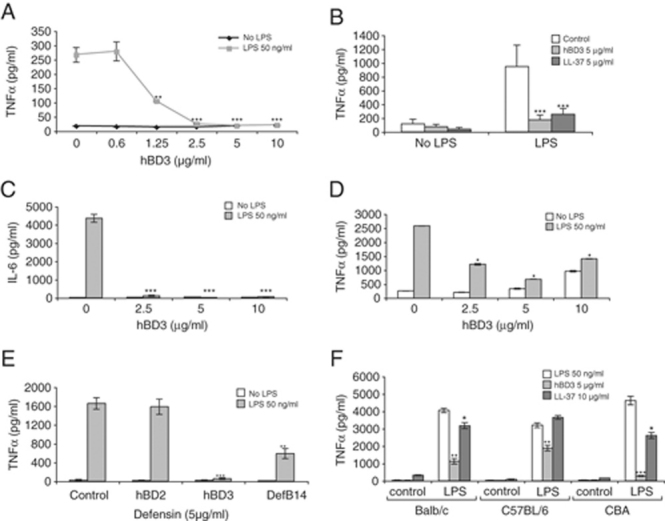
hBD3 down-regulates cytokine production in response to the TLR4 agonist LPS in human and mouse Mφ. (A) THP-1 monocyte cell line, (B) human monocyte-derived Mφ, (C and D) RAW264.7 mouse Mφ cell line and (E) BMDM from CBA mice were exposed to β-defensin or control peptides at 5 μg/mL, in the presence or absence of LPS for 18 h in serum-free media. TNF-α (or IL-6) in the supernatant was measured by ELISA, *n*=6 donors for the human primary cells, *n*=3 THP-1, *n*=3 RAW264.7 and *n*=3 mouse BMDM (from three separate mice). Figure shows means±SEM, significance assessed by two-way ANOVA, ^***^p<0.001, ^**^p<0.01 was calculated by comparing LPS plus peptide to LPS alone. (F) TNF-α levels in supernatant in BMDM from different mouse strains, treated with hBD3 or LL37 at indicated concentrations, with and without LPS for 18 h, *n*=3. Statistical comparisons are between LPS alone *versus* LPS with peptide in each strain, ^***^p<0.001, ^**^p<0.01, ^*^p<0.05.

In contrast to the assumed pro-inflammatory effect of hBD3 summarised above, we show here that synthetic hBD3 inhibits production of TNF-α by the human myelomonocytic cell line THP-1 in a concentration-dependent manner (Fig. 1A). The effect was maximal at 2.5 μg/mL, and comparable in magnitude to the cationic antimicrobial peptide LL37, which is a known immunomodulatory peptide 16–18. This same effect was also evident using human peripheral blood monocyte derived Mφ (Fig. 1B). Treatments did not affect cell viability as MTT assay measurements were comparable between treated cells and untreated controls.

Addition of hBD3 to the mouse Mφ cell line RAW264.7 also led to inhibition of TNF-α and IL-6 production (Fig. 1C and D). In our experimental settings hBD3 did not induce TNF-α or IL-6, in contrast to the recent report that this defensin activates monocytes and myeloid dendritic cells *via* TLR1/2, up-regulating the co-stimulatory molecules CD80, CD86 and CD40 12. We observe our anti-inflammatory effect with 5 μg/mL (∼1 μM) of synthetic hBD3 by directly measuring the attenuation of pro-inflammatory cytokine production, whereas Funderburg *et al* observe their effects on co-stimulatory molecules with 20 μg/mL of recombinant hBD3 (and do not measure pro-inflammatory cytokines). We did, however, observe a slight increase in TNF-α with hBD3 at 10 μg/mL but only in RAW264.7 cells (Fig. 1D), not primary Mφ or THP-1 cells, suggesting that in specific cells at higher concentrations of hBD3 there may also be a pro-inflammatory effect of hBD3.

The anti-inflammatory effect of both hBD3 and the mouse orthologue Defb14 19 was observed in mouse primary BM-derived Mφ (BMDM), reducing the TNF-α response to LPS (Fig. 1E). hBD2 was not an effective suppressor of the TNF-α response to LPS in mouse cells (Fig. 1E), whereas hBD3 was more effective than LL37 in all mouse strains tested (Fig. 1F). hBD2 has only approximately 30% amino acid similarity to hBD3, which may explain lack of anti-inflammatory effects. Conversely, Defb14, which is 64% identical to hBD3 20, did demonstrate anti-inflammatory activity.

**Figure 2:**
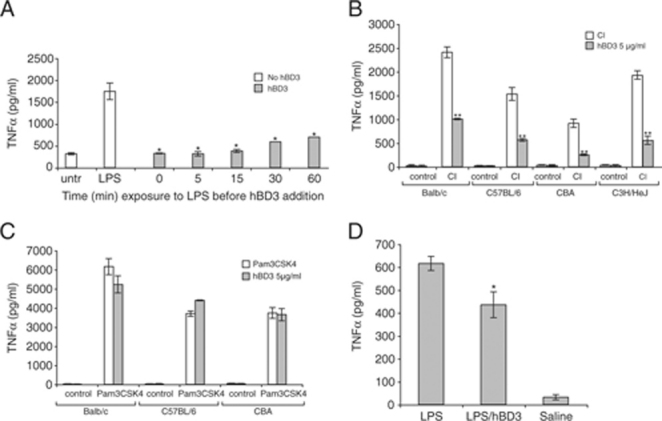
Dissection of hBD3 anti-inflammatory function and *in vivo* effects of hBD3. (A) The mouse Mφ cell line RAW 264.7 was exposed at various times up to 1 h with LPS before treatment with hBD3, followed by 18 h incubation. The inhibitory effect was still evident at all time points (*n*=2, ^*^p<0.05). (B) BMDM from different mouse strains were activated with CD40L (5 μg/mL) and IFN-γ (5 ng/mL) (CI) with and without hBD3 for 18 h. TNF-α levels in supernatants were measured. BMDM were prepared from at least three separate mice for each strain. Statistical comparisons are between agonist alone versus agonist with hBD3 within each strain, ^**^p<0.01 by two-way ANOVA. (C) TNF-α levels in supernatant in BMDM from different mouse strains, treated with hBD3 at indicated concentrations, with and without Pam_3_Csk_4_ for 18 h, *n*=3. (D) Balb/c male mice were injected i.p. with 16 mg/kg LPS with or without 10 μg hBD3 in 200 μL saline. After 1 h animals were killed and exsanguinated and serum TNF-α measured. Figure shows means±SEM and comparison between LPS alone (*n*=18) and LPS with hBD3 (*n*=21) is statistically different ^*^p=0.032 by unpaired *t*-test.

The anti-endotoxic effects of LL37 have been shown to be partly due to direct binding of LL37 to LPS 16, 21. It has previously been shown that hBD3 does not inhibit endotoxin binding in a Limulus assay 22 and we confirmed this finding (Supporting Information) to demonstrate similar endotoxin activity in the presence and absence of hBD3. However, the Limulus assay is not a direct measure of LPS-hBD3 binding; so we also investigated hBD3 effects after LPS stimulation of cells. Figure 2A shows that TNF-α levels were significantly reduced even when hBD3 was added to Mφ 1 h after LPS. This suggests that even if hBD3 binds LPS to some extent, most of the hBD3 inhibitory effect is occurring downstream of TLR4 activation by LPS.

Further evidence that hBD3 is endowed with general anti-inflammatory properties is shown in Fig. 2B. Stimulation with IFN-γ and CD40L results in Mφ activation and increased TNF-α, but here we show that hBD3 inhibited this pro-inflammatory cytokine response in mouse BMDM. This effect was also evident in C3H/HeJ Mφ, which lack functional TLR4, demonstrating that hBD3 is not simply inhibiting stimulation by endotoxin contamination. The anti-inflammatory effect was not evident when cells were exposed to PAM_3_CSK_4_ a TLR1/2 agonist (Fig. 2C). This suggests that hBD3 has an effect on signalling molecules that are used by TLR4 and CD40 but not TLR1/2. This differs from LL-37, which has been shown to inhibit pro-inflammatory responses *via* both TLR4 and TLR1/2. 16. As TLR4 and TLR1/2 signalling both involve MyD88 it is possible that hBD3 is affecting components of the non-MyD88 pathway (such as TRAM and TRIF) downstream of TLR4.

### hBD3 is anti-inflammatory *in vivo*

Next, we wished to see whether hBD3 could reduce the accumulation of TNF-α in mice following exposure to LPS. We injected 16 mg/kg LPS into male Balb/c mice with and without 10 μg of hBD3 and measured serum TNF-α levels 1 h later. We found that the group injected with hBD3 and LPS had significantly reduced levels of TNF-α compared with mice receiving LPS alone (Fig. 2D). This result demonstrates that hBD3 inhibits LPS-stimulated TNF-α production *in vivo* as well as *in vitro.* The extent of inhibition afforded by hBD3 was comparable to that conferred by 1 μg IL-10, which protects mice from endotoxic shock 23, so hBD3 may provide similar protection.

### Melanocortin receptors are not involved in hBD3 anti-inflammatory function

**Figure 3:**
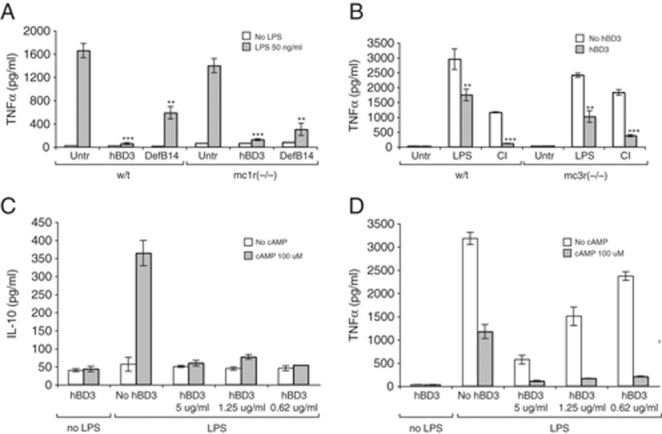
hBD3 anti-inflammatory effect does not act through MC1R or MC3R and has mechanisms distinct from cAMP. BMDM from (A) MC1R mutant and (B) MC3R mutant were stimulated with LPS alone or LPS in combination with hBD3 or DefB14 (5 μg/mL) or (B only) CD40/IFN-γ (CI) alone or in combination with hBD3 as described. After 18 h, concentrations of TNF-α in the supernatants were measured, *n*=3. Statistical comparisons are between LPS alone *versus* LPS with peptide in each strain, ^***^p<0.001, ^**^p<0.01 by two-way ANOVA. BMDM from Balb/c mice were cultured with LPS (50 ng/mL) in the presence of 8Br-cAMP (100 μM) and decreasing concentrations of hBD3, as shown, for 18 h. Concentrations of (C) IL-10 and (D) TNF-α in the supernatants were measured by ELISA.

hBD3 is a promiscuous ligand which interacts with CCR6 and another unknown Mφ receptor 14, 24. In addition, Candille *et al* elegantly show that overexpressing the dog orthologue of hBD3 alters hair colour in transgenic mice *via* binding to murine MC1R 14. We tested whether hBD3 might mediate its anti-inflammatory effect through MC1R or MC3R, as these receptors are expressed in Mφ, and the known ligand α-melanocortin stimulating hormone is an anti-inflammatory mediator 25. The absence of either receptor has also been reported to influence the response to inflammatory agents 26, 27. We tested the naturally defective *Mc1r* mutant mouse strain (recessive yellow *Mc1r^e^*) 28 and an *Mc3r* knockout mouse 29. We found no statistically significant difference between the ability of hBD3 to reduce TNF-α levels following stimulation of TLR4 or CD40 in BMDM from WT controls or mutant mice (Fig. 3A and B). This demonstrates that the anti-inflammatory properties of hBD3 are not mediated by MC1R or MC3R.

### hBD3 Anti-inflammatory effect is not mediated by IL-10 or cAMP

IL-10 is a well-known anti-inflammatory cytokine that inhibits co-stimulatory molecule expression on Mφ and limits the production of pro-inflammatory cytokines and chemokines 30. We investigated the ability of hBD3 to induce IL-10 in BMDM and established that IL-10 levels were not altered by hBD3 in the presence or absence of LPS (Fig. 3C), suggesting that the hBD3 anti-inflammatory effect is not mediated by IL-10.

cAMP is an important controller of the innate immune system, with a wide range of functions including up-regulation of IL-10 and reduction of TNF-α 31. Using the membrane permeable cAMP analogue, 8-Bromoadenosine-cAMP (8Br-cAMP), we examined similarities between cAMP and hBD3 anti-inflammatory activity. TNF-α levels induced by LPS were markedly reduced by 8Br-cAMP or hBD3 alone, however a combination of 8Br-cAMP and hBD3 reduced TNF-α levels further. This effect was evident at low concentrations of hBD3, where hBD3 alone shows minimal inhibition of TNF-α (Fig. 3D). Similarly induction of IL-10 by 8Br-cAMP was inhibited by hBD3 (Fig. 3C). These results suggest that cAMP and hBD3 act through distinct mechanisms.

## Concluding remarks

In conclusion, hBD3 is a potent inhibitor of the accumulation of pro-inflammatory cytokines TNF-α and IL-6, secreted in response to the TLR4 agonist LPS and following activation with CD40L. This effect was not due to direct peptide binding of LPS and was not mediated through the anti-inflammatory receptors MC1R or MC3R. In support of this finding hBD3 anti-inflammatory action was independent of cAMP levels and not controlled by an increase in IL-10. In addition, administration of hBD3 to mice reduced LPS-induced serum levels of TNF-α, indicating that hBD3 may be important in controlling inflammation and septic shock. The copy number variation of β-defensins at the 8p23 cluster may lead to subtle variation in expression levels in the human population 2. Up-regulation of hBD3 is critical to antimicrobial activity in the epithelia; however, the novel hBD3 functions presented here suggest a role in the resolution of inflammation, which is necessary to avoid tissue damage by effectors of antimicrobial action.

## Materials and methods

### Reagents

Ultra pure LPS from *E. coli* 0111:B4, Pam_3_CSK_4_ and IFN-γ were purchased from InvivoGen (San Diego, USA), pertussis toxin, polymixin B and 8Br-cAMP (B7880) from Sigma Dorset, UK and QCL-1000® Endpoint Chromogenic LAL Assay from Lonza Group, Basel, Switzerland. Mouse CD40L was kindly provided by Dr. David Gray (University of Edinburgh). hBD3 (GIINTLQKYYCRVRGGRCAVLSCLPKEEQIGKCSTRGRKCCRRKK) and hBD2 (GIGDPVTCLKSGAICHPVFCPRRYKQIGTCGLPGTKCCKKP) were purchased from Peptides International Louisville, USA and are oxidised so the disulfide connectivities are of the canonical β-defensin arrangement 32. Defb14 (FLPKTLRKFFCRIRGGRCAVLNCLGKEEQIGIRCSNSGRKCCRKKK) and LL37 (LLGDFFRKSKEKIGKEFKRIVQRIKDFLRNLVPRTES) were synthesized as previously described 20, 33.

### Cells and mice

RAW264.7 cells were maintained in DMEM (GIBCO Paisley, UK) and THP-1 cells in RPMI containing 10% FBS, essential amino acids and antibiotics. Balb/c, CBA and C57 Black/6 mice were obtained from Charles River (UK) and *Mc1r e/e* and *Mc3r* KO mutants were bred in-house. C3H/HeJ OlaHsd-Tlr4 mutants and C3H/HeN controls were obtained from Harlan Laboratories, UK. Primary Mφ were generated from femur BM and grown in DMEM containing 10% FBS and 20 ng/mL M-CSF (R&D Systems, Abingdon, UK) for 7 days. Cells were seeded at 1.25×10^5^ into 48-well plates and grown without growth factor for 24 h prior to treatment. Replicate experiments were done with separate Mφ preparations from at least three mice for each experiment.

### Human PBMC preparation

Human venous blood was collected according to Lothian Research Ethics Committee approvals ♯08/S1103/38, using sodium citrate anticoagulant (Phoenix Pharma, Gloucester, UK), and cells were separated by Dextran sedimentation, followed by discontinuous, isotonic Percoll gradient centrifugation as previously described 33. PBMC were incubated at 4×10^6^/mL in IMDM (PAA Laboratories, Somerset, UK) at 37°C, 5% CO_2_, for 1 h. Non-adherent cells were removed and adherent monocytes cultured for 6 days in IMDM with 10% autologous serum to generate monocyte-derived Mφ.

### Cell treatment and ELISA

Cells were treated with LPS (50 ng/mL), Pam_3_CSK_4_ (100 ng/mL), CD40L (3 μg/mL) IFN-γ (5 ng/mL), hBD3, Defb14, LL-37, 8Br-cAMP (at concentrations shown) or combinations of these as described, in serum free media then incubated at 37°C, 5% CO_2_ for 18 h. Supernatants were collected and centrifuged to remove particulate debris. Levels of TNF-α, IL-6 and IL-10 in the supernatants were measured using human or mouse DuoSet ELISA (R&D Systems) according to the manufacturer's instructions. Cell viability was measured using TACS™ MTT assay (R&D Systems).

### LPS delivery *in vivo*

Balb/c male mice (5–8 wk) were injected with 16 mg/kg of LPS (approx. 200 μg/mouse) with or without 10 μg of hBD3 in 200 μL of PBS. After 1 h mice were killed by cervical dislocation, exsanguinated and serum TNF-α levels measured by ELISA. All experiments were covered by Project License PPL 60/3787 granted by the Home Office under the Animal Scientific Procedures Act 1986, and locally approved by the University of Edinburgh Ethical Review Committee.

### Statistical analysis

GraphPad Prism 5 statistical software was used to determine statistical significance. One or two-way ANOVA with Bonferroni's multiple comparison post-tests were performed. Where appropriate, statistical significance was determined by an unpaired *t*-test using GraphPad software. For all statistical analyses *p*<0.05 was considered significant. Values are expressed as mean±SEM.

## Abbreviations

8Br-cAMP: 8-Bromoadenosine-cAMP
BMDM: BM-derived Mφ
CD40L: CD40 ligand
hBD3: human β defensin-3
MC1R: melanocortin 1 receptor
MC3R: melanocortin 3 receptor
