# Supplementary material for: Human β-defensin 3 has immunosuppressive activity in vitro and in vivo
Source: Eur J Immunol. 2010 Jan 26;40(4):1073–8. doi: 10.1002/eji.200940041 (PMC2948537; doi:10.1002/eji.200940041)
Supplement: Supplementary file 1 [file eji0040-1073-SD1.pdf]

European Journal of Immunology

Supporting Information

for

DOI 10.1002/eji.200940041

Human β-defensin 3 has immunosuppressive activity *in vitro* and *in vivo*

Fiona Semple, Shelia Webb, Hsin-Ni Li, Hetal B. Patel, Mauro Perretti, Ian J. Jackson, Mohini Gray, Donald J. Davidson and Julia R. Dorin

© 2010 WILEY-VCH Verlag GmbH & Co. KGaA, Weinheim

A

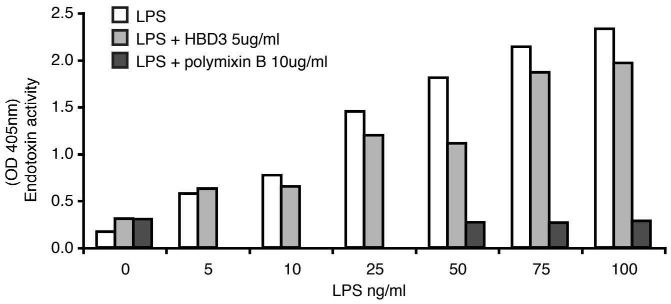

Supplementary Figure. hBD3 does not inhibit LPS endotoxin activity in the Limulus assay. hBD3 (5μg/ml) was incubated with increasing concentrations of LPS for 2hrs at 37°C. The concentration of free endotoxin in the hBD3/LPS mixture was evaluated by comparing with LPS alone in a Limulus assay.
